# Lower preoperative serum uric acid level may be a risk factor for postoperative delirium in older patients undergoing hip fracture surgery: a matched retrospective case-control study

**DOI:** 10.1186/s12871-022-01824-0

**Published:** 2022-09-07

**Authors:** Lin Xu, Wenyuan Lyu, Penghui Wei, Qiang Zheng, Chengwei Li, Zheng Zhang, Jianjun Li

**Affiliations:** 1 Department of Anesthesiology, Qilu Hospital (Qingdao), Cheeloo College of Medicine, Shandong University, Qingdao, 266035 P.R. China; 2 Department of Anesthesiology, Qilu Hospital of Shandong University, Jinan, 250000 P.R. China

**Keywords:** Hip fracture, Older people, Postoperative delirium, Uric acid, Oxidative stress, Adverse outcomes

## Abstract

**Background:**

Postoperative delirium (POD) is a common complication after hip fracture surgery that is associated with various short- and long-term outcomes. The mechanism of POD may be associated with the oxidative stress process. Uric acid has been shown to provide a neuroprotective effect in various neurodegenerative diseases through its antioxidant properties. However, it is unclear whether lower preoperative serum uric acid levels are associated with the development of POD after hip fracture surgery. Therefore, this study assessed the association of lower preoperative uric acid levels in patients with POD during hospitalization.

**Methods:**

This is a matched retrospective case-control study that included 96 older patients (≥65 y) who underwent hip fracture surgery. POD was diagnosed using the Diagnostic and Statistical Manual of Mental Disorders, Fifth Edition. Patients diagnosed with POD (cases) were matched 1:1 with patients without POD (controls) on the basis of age, sex, and anesthesia type. The relationship between preoperative uric acid and POD was analyzed by multivariable analysis.

**Results:**

The POD and non-POD groups each had 48 patients. In the univariate analysis, lower log preoperative serum uric acid value, higher neutrophil-to-lymphocyte ratio, and cerebrovascular disease were more likely in patients with POD than in those with no POD. Multivariable conditional logistic regression analysis showed that lower log preoperative serum uric acid (adjusted odds ratio [aOR], 0.028; confidence interval [CI], 0.001–0.844; *p* = 0.040), higher neutrophil-to-lymphocyte ratio (aOR, 1.314; 95% CI, 1.053–1.638; *p* = 0.015), and increased surgery duration (aOR, 1.034; 95% CI, 1.004–1.065; *p* = 0.024) were associated with increased risk of POD.

**Conclusions:**

Lower preoperative serum uric acid levels may be an independent risk factor for POD after adjustment for possible confounding factors. However, large prospective studies are needed to confirm this finding.

## Background

Hip fracture constitutes a major health problem in older patients. Postoperative delirium (POD) is a common complication in older patients after hip fracture surgery, with an incidence of 13.0–55.9 % [1]. POD is associated with poor outcomes, including prolonged hospital stay, higher costs, and increased mortality [2–4]. Moreover, POD may be associated with long-term cognitive and functional decline [5, 6]. Hip fracture is becoming a major public health concern owing to the increasing life expectancy of the global population and subsequent aging of the demographic structure [7]; therefore, it is important to understand the risk factors for POD.

Inflammation and oxidative stress after hip surgery may be involved in the pathophysiology of POD [6]. Uric acid (UA) is a natural antioxidant, and reportedly has a neuroprotective impact in various neurodegenerative diseases, including Parkinson's disease, Alzheimer's disease, and multiple sclerosis [8–10]. Its antioxidant properties may protect against the detrimental effect of oxidative stress in people with central nervous system disorders [10–12]. However, it is unclear whether lower preoperative serum UA (SUA) levels are associated with the development of POD in older patients following hip fracture surgery. The aim of this retrospective case-control study was to explore the relationship between preoperative SUA levels and POD in older patients who underwent hip fracture surgery.

## Methods

### Study design and patient selection

This retrospective, matched, case-controlled study was conducted at Qilu Hospital (Qingdao), Cheeloo College of Medicine, Shandong University, Qingdao, P.R. China, a tertiary academic center. Ethical approval was obtained from the Medical Ethics Committee of Qilu Hospital of Shandong University (Qingdao) (KYLL-202205). The requirement for written informed consent from each patient was waived because of the study's retrospective manner, and the Medical Ethics Committee of Qilu Hospital of Shandong University (Qingdao) approved the waiver. Ethics approval was obtained on February 24, 2022, and data collection commenced in March 1, 2022. This study followed the Strengthening the Reporting of Observational Studies in Epidemiology (STROBE) guidelines.

We retrospectively searched electronic health records for older patients with recent fractures (less than 3 weeks at the time of operation) who underwent hip fracture surgery between May 1, 2020, and May 1, 2021 at Qilu Hospital (Qingdao). The inclusion criteria for patients in this study were as follows: age ≥ 65 years; patients underwent elective surgery; all patients provided authorization for the use of their medical records for research purposes. We excluded patients diagnosed with severe renal insufficiency or renal failure, patients with gout, patients who were taking drugs that affect SUA level or anti-Parkinson drugs that may induce hallucinations, and participants with missing data.

### Retrospective delirium ascertainment and case-control matching

POD was diagnosed based on a retrospective chart review method [13]. All medical and nursing records were carefully reviewed to assess delirium. Patients were included in the POD group if the case notes suggested that they were diagnosed with delirium according to the Diagnostic and Statistical Manual of Mental Disorders, Fifth Edition (DSM-V, 2013), or billed for antipsychotics (specifically olanzapine, haloperidol, and quetiapine) [14]. POD was diagnosed by neurologists at any postoperative stage, or by three experienced anesthesiologists during review of the electronic medical records. For those who were not assessed by neurologists, the patient was included in the POD group when two or more evaluators reached consensus on the diagnosis of delirium. Referring to the study by Guldolf K, et al [15], Table 1 shows the abstract symptoms and reported comments related to the specific DSM-5 criteria. Patients diagnosed with POD (cases) were matched 1:1 with patients without POD (controls) on the basis of age (± one year), sex, and anesthesia type.

**Table 1:** Abstracted symptoms in relation to the DSM-5 criteria

DSM-5 criteria		Abstracted symptoms
A.	A disturbance in attention (i.e., reduced ability to direct, focus, sustain, and shift attention) and awareness (reduced orientation to the environment).	Any verbatim comment e.g. "agitated", "drowsiness", "somnolent", "not alert", "slept poorly", "fumbles", "pulls out urine probe", "tears off wound dressing" Any formal rating e.g. GCS, RASS
B.	The disturbance develops over a short period of time (usually hours to a few days), represents a change from baseline attention and awareness, and tends to fluctuate in severity during the day.	Any verbatim comment indicating a change in mental state which was recovered in short time after treatment.
C.	An additional disturbance in cognition (e.g. memory deficit, disorientation, language, visuospatial ability, or perception).	Any comment e.g. "confused", "disorientated", "talking nonsense", "hallucinations"
D.	The disturbances in Criteria A and C are not better explained by a pre-existing, established or evolving neurocognitive disorder and do not occur in the context of a severely reduced level of arousal, such as coma.	Any formal assessment e.g. GCS, RASS; any formal cognitive assessment e.g. AMT, MMSE Any formal specialty assessment, e.g. neurology, geriatric medicine, liaison psychiatry Any verbatim comment such as "more confused", "comatose", "no response at all"
E.	There is evidence from the history, physical examination or laboratory findings that the disturbance is a direct physiological consequence of another medical condition, substance intoxication or withdrawal, or exposure to a toxin, or is due to multiple etiologies.	Hip fracture surgery was considered a precipitating medical condition, and was present in all patients General clinical vignette, including metabolic and laboratory parameters taken closest to date of prevalence study

*AMT* Abbreviated Mental Test, *GCS* Glasgow Coma Scale, *MMSE* Mini-Mental State Examination, *RASS* Richmond Agitation-Sedation Scale

### Data abstraction

Medical, surgical, and anesthesia records were electronically abstracted. The following variables were collected: sex, age, body mass index, and medical history (diabetes mellitus, coronary artery disease [CAD], hypertension, and cerebrovascular disease). Preoperative laboratory data (most recent results before surgery) including neutrophil count; lymphocyte count; hemoglobin concentration; erythrocyte sedimentation rate; and albumin, C-reactive protein, SUA, blood urea nitrogen, creatinine, aspartate transferase, and alanine transferase levels.

During the perioperative period, we recorded the surgery and anesthesia duration, intraoperative medications, intraoperative fluid administration and blood transfusion, and intraoperative blood loss and urine output. After surgery, postoperative complications including the duration of hospital stay, pneumonia, and thrombosis were recorded.

### Bias

Bias is an unavoidable drawback in retrospective studies. However, we fully considered this issue and took measures to reduce offsets. To be diagnosed with POD, the medical course and records had to demonstrate clear documentation of clinically manifest delirium in the form of DSM-V. Each of the two anesthesiologists collected the data of all consecutive patients, and the data gathered by each author was compared to ensure accuracy. To reduce the potential biases associated with missing data of variables, we used only items that appeared in all patients’ laboratory data. Group allocation was performed after data collection, thereby reducing bias. Multivariate logistic regression analysis was used to adjust for confounders.

### Statistical analysis

Data were summarized as means (standard deviations) or medians (interquartile range) for continuous variables, and frequencies (percentage) for nominal variables. To identify differences between POD and non-POD groups, the distribution of the data was assessed using the Shapiro–Wilk test, continuous variables were assessed with the appropriate two-sample method (paired t-test or rank-sum test), and categorical variables were assessed with the chi-squared or Fisher’s exact tests. Two-tailed p-values are reported, and *p* < 0.05 was considered statistically significant. Levels of preoperative SUA were normalized by log_10_ transformation. Conditional logistic regression analysis was used to assess variables independently associated with POD, and adjusted odds ratios with 95% CIs were calculated. To avoid overestimation, a conservative approach was used: all variables with *p* < 0.20 on univariate analyses (including a history of cerebrovascular disease and CAD, log preoperative SUA, dezocine, infusion volume, duration of surgery, and neutrophil-to-lymphocyte ratio [NLR] value) were included [16]. All analyses were performed using SPSS 25 software (IBM Corp., Armonk, NY, USA).

## Results

### Demographic data and baseline characteristics of patients

**Fig. 1:**
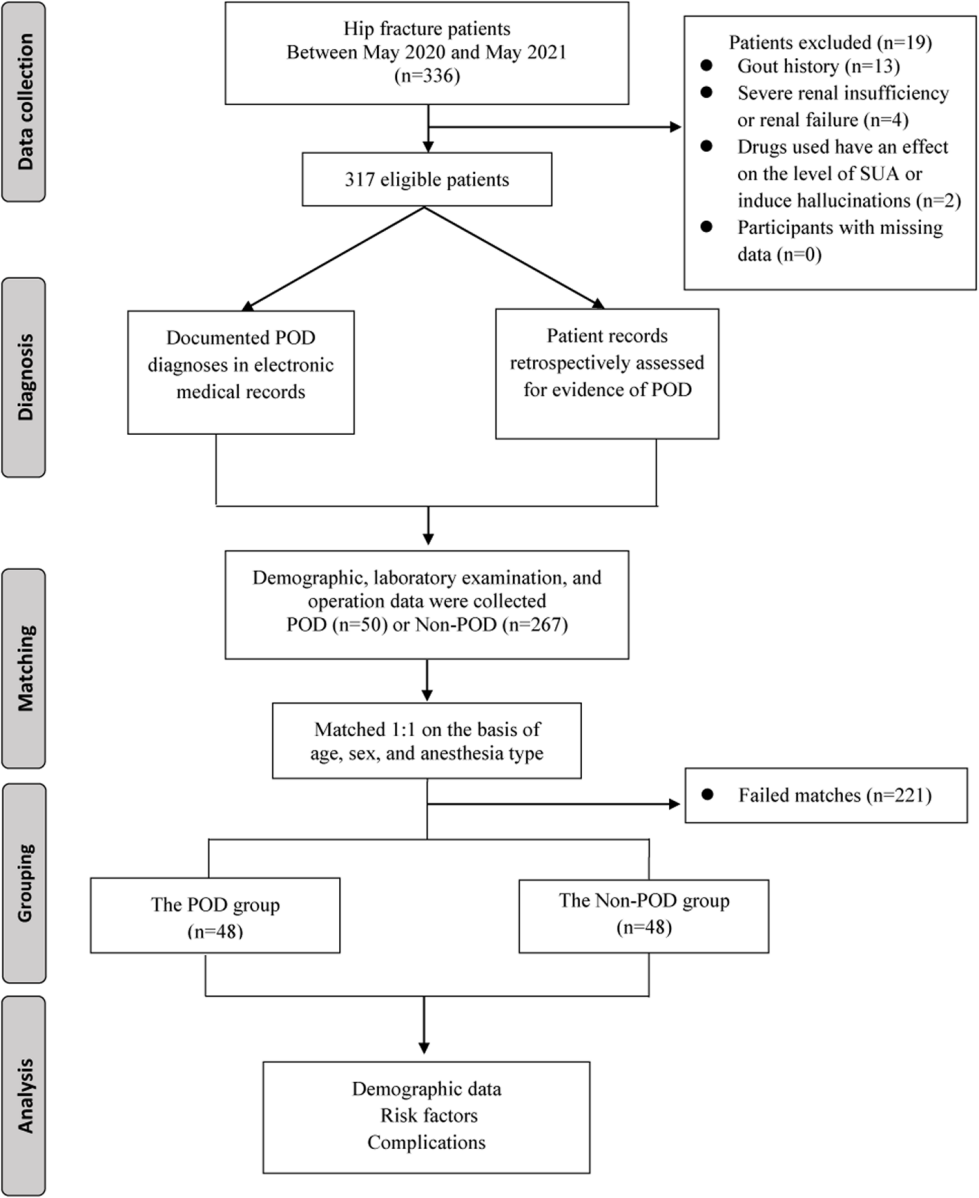
Flowchart of patient identification and delirium diagnosis

During the study period, a total of 336 patients were identified. Nineteen patients were excluded on the basis of a history of gout (13), severe renal insufficiency or renal failure (4), drugs influenced the level of SUA or induced hallucinations (2), and participants with missing data (0). A total of 317 patients were eligible, of whom 50 patients were diagnosed with delirium, for an incidence of 15.8%. Patients who failed to match were further excluded (221), and a total of 96 patients were included in the analysis. Figure 1 shows the flowchart of patient data inclusion within this study. Patients diagnosed with POD (*n* = 48) were matched 1:1 with patients without POD (*n* = 48) on the basis of age, sex, and anesthesia type. Of the 96 patients, 80 (83.3%) were female. The median age of the POD group was 86.5 years (interquartile range, 81.3, 90.0), and that of the non-POD group was 86.5 years (interquartile range, 81.0, 90.0). The number of patients who underwent combined spinal-epidural anesthesia was 40 (83.33%) in both groups (Table 2).

**Table 2:** Patient characteristics and anesthetic types used to match cases and controls

Variable	Case (*n*=48)	Control (*n*=48)
**Age, year, M (*P***_***25,***_ ***P***_***75***_**)**	86.50 (81.25,90.00)	86.50 (81.00,90.00)
**Sex, n (%)**		
Male	8 (16.67)	8 (16.67)
Female	40 (83.33)	40 (83.33)
**Anesthetic modes, n (%)**		
CSEA	40 (83.33)	40 (83.33)
GA	8 (16.67)	8 (16.67)

Data are presented as medians (interquartile range) or number (%). *Abbreviations*: *CSEA* Combined spinal-epidural anesthesia, *GA* General anesthesia

### Risk factor analysis of POD

In the univariate analyses (Table 3), the following factors were associated with the occurrence of POD: history of cerebrovascular disease, including stroke and transient ischemic attack (15 [31.25%] vs. 6 [12.50%], *p* = 0.039), log preoperative SUA (2.33 ± 0.16 vs. 2.42 ± 0.13, *p* = 0.015), and NLR (5.24 [3.27,8.34] vs. 3.94 [2.70,5.30], *p* = 0.007).

**Table 3:** Analysis of risk factors for POD

	Univariate analysis			Multivariate analysis	
	Case (*n*=48)	Control (*n*=48)	*p* value	aOR (95% CI)	*p* value
**BMI, (kg/m2)**					
Underweight	6 (12.50)	2 (4.17)	.312		
Normal	23 (47.92)	23 (47.92)	Ref		
Overweight	12 (25.00)	20 (41.67)	.292		
Obesity	7 (14.58)	3 (6.25)	.270		
**Medical history, n (%)**					
Diabetes mellitus	12 (25.00)	11 (22.92)	.808		
Hypertension	32 (66.67)	30 (62.50)	.566		
Cerebrovascular disease	15 (31.25)	6 (12.50)	.039	1.594 (0.454-5.596)	.467
CAD	20 (41.67)	13 (27.08)	.134	1.734 (0.457-6.577)	.419
**Pre. Labo data**					
Cr, mg/dL	63.00 (50.00,83.00)	62.00 (52.25,76.75)	.463		
BUN, mg/dL	7.20 (4.93,9.08)	6.65 (4.80,8.48)	.297		
Log preoperative SUA^a^	2.33 ± 0.16	2.42 ± 0.13	.015	0.028 (0.001-0.844)	.040
ALT, U/L	12.50 (9.00,16.00)	10.00 (8.00,18.50)	.651		
AST, U/L	17.00 (14.25,20.75)	18.00 (15.25,23.00)	.829		
**Intraoperative medications, n (%)**					
Dexmedetomidine	35 (72.92)	37 (77.08)	.638		
Midazolam	1 (2.08)	2 (4.17)	.571		
Droperidol	2 (4.17)	1 (2.08)	.571		
Butorphanol	6 (12.50)	7 (14.58)	.782		
Dexamethasone	11 (22.92)	13 (27.08)	.655		
Dynastat	3 (6.25)	5 (10.42)	.484		
Dezocine	2 (4.17)	6 (12.50)	.178	0.525 (0.058-4.771)	.567
**Intraoperative factors**					
Infusion volume, mL	800.0 (700.0,1000.0)	800. 0 (600.0,1000.0)	.091	1.002 (0.999-1.005)	.186
Hemorrhage volume, mL	200.0 (112.5,200.0)	200.0 (118.8,222.5)	.728		
Urine output, mL	200.0 (100.0,275.0)	175.0 (100.0,200.0)	.245		
Duration of anesthesia, min	65.50 (52.25,79.25)	57.00 (50.25,70.50)	.483		
Duration of surgery, min	116.21 ± 26.59	112.88 ± 23.28	.062	1.034 (1.004-1.065)	.024
**Blood RT**					
ESR, mm/h	36.75 ± 20.54	35.00 ± 17.29	.649		
NLR	5.24 (3.27,8.34)	3.94 (2.70,5.30)	.007	1.314 (1.053-1.638)	.015
Hb, g/dL	101.94 ± 18.28	106.98 ± 23.02	.269		
CRP, mg/L	61.10 (46.22,97.72)	63.92 (38.12,94.43)	.292		
ALB, g/L	35.91 ± 3.81	36.80 ± 3.75	.217		
Cys C, mg/L	0.78 (0.58,0.99)	0.80 (0.65,1.06)	.695		
Blood glucose, mmol/L	5.98 (5.07,7.18)	5.64 (5.14,6.97)	.730		
**Other risk factors**					
Electrolyte disorder, n (%)	21 (43.75)	18 (37.50)	.549		

^a^Levels of preoperative SUA were normalized by log_10_ transformation

Data are presented as number (%), means (standard deviations), or medians (interquartile range). *Abbreviations*: *ALB* Albumin, *ALT* Alanine transaminases, *AST* Aspartate aminotransferases, *aOR* Adjusted odds ratio, *Blood RT* Blood routine test, *BMI* Body Mass Index, *BUN* Blood urea nitrogen, *CAD* Coronary artery disease, *CI* Confidence interval, *CRP* C-reactive protein, *Cr* Creatinine, *Cys C* Cystatin C, *ESR* Erythrocyte sedimentation rate, *Hb* Hemoglobin, *NLR* Neutrophil-to-lymphocyte ratio, *SUA* Serum uric acid

Multivariable analysis (Table 3) showed three independent risk factors for the occurrence of POD, including log preoperative SUA (adjusted odds ratio [aOR], 0.028; 95% CI, 0.001–0.844; *p* = 0.040), NLR (aOR, 1.314; 95% CI, 1.053–1.638; *p* = 0.015), and surgery duration (aOR, 1.034; 95% CI, 1.004–1.065; *p* = 0.024) (Table 3).

### Postoperative complications

In our study, the length of hospital stay in the POD group was significantly longer than that in the non-POD group (10.90 [8.0,12.0] vs. 8.27 [7.0,9.0], *p* < .001, Table 4). However, there was no significant difference in the incidence of pneumonia or thrombosis between the two groups.

**Table 4:** Outcomes: cases vs matched controls

Outcomes	Case (*n*=48)	Control (*n*=48)	t/χ2	*p* value
Length of stay, day^a^	10.86 ± 5.15	8.32 ± 2.63	-3.798	<.001
Hypostatic pneumonia^b^	12 (25.00)	8 (16.67)	1.011	.315
Thrombosis^b^	13 (27.08)	12 (25.00)	.054	.816

Data are presented as mean ± SD and number (%)

^a^paired t-test, ^b^chi-square test

## Discussion

In this retrospective case-control study, we found that a lower preoperative SUA level was associated with a higher risk of POD. A higher NLR value and increased duration of surgery were also identified as independent risk factors of POD in older patients after hip fracture. The incidence of POD after hip fracture surgery was 15.8% in our study.

A recent study reported the neuroprotective potential of UA in animal models and clinical trials [17]. Moreover, epidemiological data have indicated an inverse association between UA and several neurological disorders. An emerging body of evidence has indicated that lower SUA levels are associated with poorer cognitive function and increased risk of mild cognitive impairment [12, 18]. Furthermore, a decreased SUA level is related to a markedly higher risk of progressing to Alzheimer's disease dementia from a non-demented state [19, 20], and the development of nonmotor symptoms in patients with Parkinson's disease [10]. No previous study has investigated the correlation between preoperative SUA levels and POD; we found that a lower preoperative SUA level was an independent risk factor for POD in older patients with hip fracture surgery.

The exact underlying pathophysiology of POD is elusive and several biological models of delirium have been proposed, including neuro-inflammation, neuro-aging, neuro-endocrine stress, neurotransmitter dysregulation, oxidative stress, sleep/wake dysregulation, and network dysconnectivity [21]. The widely accepted theory is the “oxidative stress hypothesis,” which states that cerebral tissue is susceptible to oxidative damage because of its high oxygen requirement for metabolism, high level of polyunsaturated fatty acid, and low concentration of antioxidant resources [22]. As a natural antioxidant, the antioxidant properties of UA include the capacity to neutralize and scavenge prooxidant molecules, such as hydroxyl radicals, hydrogen peroxide, and peroxynitrite, and to suppress the Fenton reaction, chelate transition metals, and prevent lipid peroxidation [23, 24]. Moreover, several studies showed that in in vitro cultures urate markedly enhanced survival of dopaminergic neurons in a model of spontaneous cell death. The protective effects of urate have also been found in vivo, and *URAT1* and *GLUT9* expressed in mouse astrocytes reportedly protect dopaminergic neurons from cellular oxidative stress through intracellular accumulation of UA [25]. Thus, based on the biochemical ability of UA, there is indeed evidence for the neuroprotective role of SUA.

We also found that increased duration of surgery and NLR could serve as independent risk factors for POD in older patients undergoing hip fracture surgery. Numerous studies have established operation time as a risk factor for POD in various types of surgical procedures [26, 27]. There is no doubt that longer surgery duration indicates a more complex procedure with severe surgical stimulation and may result in greater acute inflammatory responses [27, 28]. The neuroinflammatory hypothesis is a widely accepted explanation for the development of POD [29]. It has been hypothesized that acute peripheral inflammatory stimulation, brain parenchymal cell activation, and proinflammatory cytokine expression could lead to neuronal cell apoptosis and synaptic dysfunction [29]. NLR, as a reliable measure of systemic inflammation, has been shown to be a good predictor of outcomes for neurological and psychiatric disorders in previous studies [30, 31]. The close association between inflammation and POD may be a possible explanation for the relationship between elevated NLR values and POD in our study. Notably, the trauma may have already initiated the inflammatory cascade in patients with hip fracture. It is likely that increasing inflammatory mediators induced by anesthesia-surgical intervention contribute to the risk of POD [32].

Consistent with previous studies, our analyses revealed a longer length of hospital stay for patients with POD. Prolonged length of stay can delay the patient’s ability to recover their nutritional status [33]. However, there was no significant difference in the incidence of pneumonia and thrombosis between the POD and non-POD groups in our analysis. The causes of pneumonia and thrombosis are complex and include not only prolonged recumbency, but also the location and duration of surgery, and pre-existing underlying diseases. A multidisciplinary approach to develop a reasonable surgical plan may reduce the incidence of postoperative complications.

Although the results of this study were promising, there are some limitations. The major limitation is the bias caused by the retrospective design. We did, however, use a matched study to minimize the effect of confounding factors such as selection bias. Pairing characteristics were carefully selected, including age, sex, and anesthesia type, which have a strong relationship with POD [34–37]. We improved upon a chart-based method which had been previously validated for identifying POD from medical records [13]. A retrospective study published in *European Journal of Anaesthesiology* showed a 15.7% incidence of POD [14], which was similar to our findings. This supports the idea that delirium can be assessed retrospectively with good accuracy, and the retrospective chart review method is more appropriate for the clinical situation and retrospective research application. Another limitation is that the lack of control over relevant confounding factors (i.e., dementia and cognitive impairment), and the small sample size may affect the accuracy of the interpretation. Our results should thus be interpreted cautiously, and a prospective study with a larger sample size is required.

## Conclusions

Currently, POD remains an entirely clinical diagnosis with no established biomarkers to guide the diagnosis or management. Our observation of a significant association between preoperative SUA and POD is suggestive. Whether this represents a causal relationship remains to be determined. In summary, our study indicates a potential relationship between low preoperative SUA levels and POD among elderly patients. However, a large prospective study is required to identify whether preoperative SUA level could serve as a risk marker for POD, which could enable proactive interventions.

## Data Availability

The data that support the findings of this study are available from the corresponding author, JL, upon reasonable request.

## Abbreviations

ALB: Albumin
ALT: Alanine transaminases
AMT: Abbreviated Mental Test
AST: Aspartate aminotransferases
aOR: Adjusted odds ratio
Blood RT: Blood routine test
BMI: Body mass index
BUN: Blood urea nitrogen
CAD: Coronary artery disease
CI: Confidence interval
CRP: C-reactive protein
Cr: Creatinine
CSEA: Combined spinal-epidural anesthesia
Cys C: Cystatin C
DSM-5: Diagnostic and Statistical Manual of Mental Disorders, Fifth Edition
ESR: Erythrocyte sedimentation rate
GA: General anesthesia
GCS: Glasgow Coma Scale
Hb: Hemoglobin
MMSE: Mini-Mental State Examination
NLR: Neutrophil-to-lymphocyte ratio
POD: Postoperative delirium
RASS: Richmond Agitation-Sedation Scale
SUA: Serum uric acid
UA: Uric acid

## References

[1] Yang Y, Zhao X, Dong T, Yang Z, Zhang Q, Zhang Y (2017). Risk factors for postoperative delirium following hip fracture repair in elderly patients: a systematic review and meta-analysis. Aging Clin Exp Res..

[2] Witlox J, Eurelings LS, de Jonghe JF, Kalisvaart KJ, Eikelenboom P, van Gool WA (2010). Delirium in elderly patients and the risk of postdischarge mortality, institutionalization, and dementia: a meta-analysis. JAMA.

[3] Saczynski JS, Marcantonio ER, Quach L, Fong TG, Gross A, Inouye SK, Jones RN (2012). Cognitive trajectories after postoperative delirium. N Engl J Med.

[4] Li T, Li J, Yuan L, Wu J, Jiang C, Daniels J, Mehta RL, Wang M, Yeung J, Jackson T, Melody T, Jin S, Yao Y, Wu J, Chen J, Smith FG, Lian Q (2022). Effect of Regional vs General Anesthesia on Incidence of Postoperative Delirium in Older Patients Undergoing Hip Fracture Surgery: The RAGA Randomized Trial. JAMA.

[5] Chen XY, Dou YX, Luo DD, Zhang ZB, Li CL, Zeng HF, Su ZR, Xie JH, Lai XP, Li YC (2017). β-Patchoulene from patchouli oil protects against LPS-induced acute lung injury via suppressing NF-κB and activating Nrf2 pathways. Int Immunopharmacol.

[6] Hshieh TT, Saczynski J, Gou RY, Marcantonio E, Jones RN, Schmitt E, Cooper Z, Ayres D, Wright J, Travison TG, Inouye SK (2017). Trajectory of Functional Recovery After Postoperative Delirium in Elective Surgery. Ann Surg.

[7] Marks R (2010). Hip fracture epidemiological trends, outcomes, and risk factors, 1970-2009. Int J Gen Med.

[8] Moccia M, Lanzillo R, Palladino R (2015). Uric acid: a potential biomarker of multiple sclerosis and of its disability. Clin Chem Lab Med..

[9] Scheepers L, Jacobsson L, Kern S, Johansson L, Dehlin M, Skoog I (2019). Urate and risk of Alzheimer's disease and vascular dementia: A population-based study. Alzheimers Dement..

[10] Shi X, Zheng J, Ma J, Wang Z, Sun W, Li M, Huang S, Hu S (2022). Low serum uric acid levels are associated with the nonmotor symptoms and brain gray matter volume in Parkinson's disease. Neurol Sci.

[11] Baik K, Chung SJ, Yoo HS, Lee YH, Jung JH, Sohn YH, Lee PH (2020). Sex-dependent association of urate on the patterns of striatal dopamine depletion in Parkinson's disease. Eur J Neurol.

[12] Chen C, Li X, Lv Y, Yin Z, Zhao F, Liu Y, Li C, Ji S, Zhou J, Wei Y, Cao X, Wang J, Gu H, Lu F, Liu Z, Shi X (2021). High Blood Uric Acid Is Associated With Reduced Risks of Mild Cognitive Impairment Among Older Adults in China: A 9-Year Prospective Cohort Study. Front Aging Neurosci.

[13] Kuhn E, Du X, McGrath K (2014). Validation of a consensus method for identifying delirium from hospital records. PLoS One..

[14] Poeran J, Cozowicz C, Zubizarreta N (2020). Modifiable factors associated with postoperative delirium after hip fracture repair: An age-stratified retrospective cohort study. Eur J Anaesthesiol..

[15] Guldolf K, Vandervorst F, Gens R, Ourtani A, Scheinok T, De Raedt S (2021). Neutrophil-to-lymphocyte ratio predicts delirium after stroke. Age Ageing.

[16] Zhou ZR, Wang WW, Li Y, Jin KR, Wang XY, Wang ZW, Chen YS, Wang SJ, Hu J, Zhang HN, Huang P, Zhao GZ, Chen XX, Li B, Zhang TS (2019). In-depth mining of clinical data: the construction of clinical prediction model with R. Ann Transl Med.

[17] Petruzzo M, Moccia M (2018). Time to reconsider urate: Neuroprotective potential may prevail on cardiovascular risk in animal models and clinical trials. EBioMedicine.

[18] Liu M, Wang J, Zeng J, He Y (2017). Relationship between serum uric acid level and mild cognitive impairment in Chinese community elderly. BMC Neurol.

[19] Du N, Xu D, Hou X, Song X, Liu C, Chen Y, Wang Y, Li X (2016). Inverse Association Between Serum Uric Acid Levels and Alzheimer's Disease Risk. Mol Neurobiol.

[20] Kim JW, Byun MS, Yi D, Lee JH, Jeon SY, Ko K, Jung G, Lee HN, Lee JY, Sohn CH, Lee YS, Shin SA, Kim YK, Lee DY (2020). Serum Uric Acid, Alzheimer-Related Brain Changes, and Cognitive Impairment. Front Aging Neurosci.

[21] Maldonado JR (2013). Neuropathogenesis of delirium: review of current etiologic theories and common pathways. Am J Geriatr Psychiatry.

[22] Sultana R, Butterfield DA (2009). Oxidatively modified, mitochondria-relevant brain proteins in subjects with Alzheimer disease and mild cognitive impairment. J Bioenerg Biomembr.

[23] Davies KJ, Sevanian A, Muakkassah-Kelly SF, Hochstein P (1986). Uric acid-iron ion complexes. A new aspect of the antioxidant functions of uric acid. Biochem J.

[24] Squadrito GL, Cueto R, Splenser AE, Valavanidis A, Zhang H, Uppu RM, Pryor WA (2000). Reaction of uric acid with peroxynitrite and implications for the mechanism of neuroprotection by uric acid. Arch Biochem Biophys.

[25] Cipriani S, Desjardins CA, Burdett TC, Xu Y, Xu K, Schwarzschild MA (2012). Protection of dopaminergic cells by urate requires its accumulation in astrocytes. J Neurochem.

[26] Mu DL, Wang DX, Li LH, Shan GJ, Li J, Yu QJ, Shi CX (2010). High serum cortisol level is associated with increased risk of delirium after coronary artery bypass graft surgery: a prospective cohort study. Crit Care.

[27] Chen D, Li Y, Li Q, Gao W, Li J, Wang S, Cao J (2021). Risk Factors and a Nomogram Model Establishment for Postoperative Delirium in Elderly Patients Undergoing Arthroplasty Surgery: A Single-Center Retrospective Study. Biomed Res Int.

[28] Lin Y, Chen J, Wang Z (2012). Meta-analysis of factors which influence delirium following cardiac surgery. J Card Surg.

[29] Cerejeira J, Firmino H, Vaz-Serra A, Mukaetova-Ladinska EB (2010). The neuroinflammatory hypothesis of delirium. Acta Neuropathol.

[30] Rembach A, Watt AD, Wilson WJ, Rainey-Smith S, Ellis KA, Rowe CC, Villemagne VL, Macaulay SL, Bush AI, Martins RN, Ames D, Masters CL, Doecke JD (2014). An increased neutrophil-lymphocyte ratio in Alzheimer's disease is a function of age and is weakly correlated with neocortical amyloid accumulation. J Neuroimmunol.

[31] An P, Zhou X, Du Y, Zhao J, Song A, Liu H, Ma F, Huang G (2019). Association of Neutrophil-Lymphocyte Ratio with Mild Cognitive Impairment in Elderly Chinese Adults: A Case-control Study. Curr Alzheimer Res.

[32] Noah AM, Almghairbi D, Evley R, Moppett IK (2021). Preoperative inflammatory mediators and postoperative delirium: systematic review and meta-analysis. Br J Anaesth.

[33] Kinoshita H, Saito J, Takekawa D, Ohyama T, Kushikata T, Hirota K (2021). Availability of preoperative neutrophil-lymphocyte ratio to predict postoperative delirium after head and neck free-flap reconstruction: A retrospective study. PLoS One.

[34] Lee HB, Mears SC, Rosenberg PB, Leoutsakos JM, Gottschalk A, Sieber FE (2011). Predisposing factors for postoperative delirium after hip fracture repair in individuals with and without dementia. J Am Geriatr Soc..

[35] Aldecoa C, Bettelli G, Bilotta F (2017). European Society of Anaesthesiology evidence-based and consensus-based guideline on postoperative delirium. Eur J Anaesthesiol..

[36] Kubota K, Suzuki A, Ohde S (2018). Age is the Most Significantly Associated Risk Factor With the Development of Delirium in Patients Hospitalized for More Than Five Days in Surgical Wards: Retrospective Cohort Study. Ann Surg..

[37] Genet B, Lamy T, Cohen-Bittan J (2022). Lack of association between perioperative medication and postoperative delirium in hip fracture patients in an orthogeriatric care pathway. J Am Med Dir Assoc..

